# Cachexia and Wasting in Chronic Illness: Regulatory and Clinical Trial Update

**DOI:** 10.1002/jcsm.70128

**Published:** 2025-11-26

**Authors:** Francesco Fioretti, Stephan von Haehling, Andrew J. S. Coats, Javed Butler, Egidio Del Fabbro, Richard J. E. Skipworth, Barry J. A. Laird, Stefan D. Anker

**Affiliations:** ^1^ Baylor Scott & White Research Institute Dallas Texas USA; ^2^ Cardiology Unit ASST Spedali Civili Hospital and University of Brescia Brescia Italy; ^3^ Department of Cardiology and Pneumology University of Göttingen Medical Center Göttingen Germany; ^4^ German Center for Cardiovascular Research (DZHK), Partner Site Göttingen Göttingen Germany; ^5^ University of Warwick Coventry UK; ^6^ Heart Research Institute Sydney New South Wales Australia; ^7^ University of Mississippi Jackson Mississippi USA; ^8^ Medical College of Georgia Augusta University Augusta Georgia USA; ^9^ Clinical Surgery University of Edinburgh, Royal Infirmary of Edinburgh Edinburgh UK; ^10^ St Columba's Hospice Care Edinburgh UK; ^11^ Edinburgh Cancer Research Centre University of Edinburgh Edinburgh UK; ^12^ Department of Cardiology (CVK) of German Heart Center Charité German Centre for Cardiovascular Research (DZHK) Partner Site Berlin, Charité Universitätsmedizin Berlin Germany

**Keywords:** cachexia, clinical trial, endpoints, regulatory issues, treatment

## Abstract

Cachexia, a syndrome marked by nonintentional weight loss, muscle wasting, functional decline and poor prognosis, affects 50%–80% of cancer patients, severely impacting quality of life, treatment tolerance and survival. A ‘Regulatory and Trial Update Workshop’ was organized by the Society on Cachexia and Wasting Disorders (SCWD) in December 2024 in Washington, DC, focused on clinical trial endpoints, standards of care and recent advancements. This article provides a summary of the discussions that were held during the first day of the workshop. Despite ongoing research, effective therapies for cachexia remain limited. Existing treatments, such as nutritional supplements, progestins, anti‐inflammatories and anabolic agents, have shown mixed results, often improving appetite or lean mass without consistent functional benefits. Common muscle mass measurements, like CT scans of the L3 vertebra, are inadequate as primary endpoints because of biological variability and small effect sizes and because they do not necessarily translate into clinical benefit. Trials continue to face challenges in meeting regulatory requirements, which mandate improvements in both body composition and functional outcomes. Regulatory consensus emphasizes demonstrating clinically meaningful benefits in patient‐reported outcomes/physical function and/or morbidity–mortality using validated instruments, adequate safety exposure, recognition that handgrip and weight alone are insufficient, feasibility in advanced disease, consideration of general activity measures, optional but informative body composition data and, for a pan‐cancer label, benefits across at least three distinct cancers. Patient‐centred endpoints, emphasizing real‐life functioning and social participation, are essential as patients prioritize daily activity and independence over isolated physical measures. Clinical trials presented during the meeting included the MENAC trial, which tested a multimodal intervention combining nutrition, exercise, anti‐inflammatory drugs and cancer therapy, achieved modest weight stabilization but no significant improvements in muscle mass or activity. In contrast, TCMCB07, an MC‐4 receptor antagonist, demonstrated promising results in preclinical and early‐phase human studies, showing weight stabilization and improved caloric intake with good tolerability. ART27.13, a dual CB1/CB2 receptor agonist, also demonstrated positive effects in appetite stimulation and weight stabilization. For S‐pindolol, which targets appetite and metabolism, Phase IIb/III trials are to be initiated, following an earlier Phase II trial that showed improved muscle mass and muscle strength (hand grip strength). Future treatments must focus on integrating patient‐centred goals, therapeutic mechanisms and meaningful clinical outcomes.

## 1 Introduction

In December 2024, a ‘Regulatory and Trial Update Workshop’, organized by the Society on Cachexia and Wasting Disorders (SCWD), was held in Washington, DC, with experts from academia, industry and the US Food and Drug Administration (FDA) attending. This article summarizes the key themes of 5 December 2024, the first day of the workshop, highlighting discussion on background and history, standard of care, trial endpoints in cachexia research and regulatory issues in the approval of cachexia treatments.

## 2 Background and History

Cachexia is a high medical need condition associated with many chronic illnesses. It is associated with poor health outcomes in all populations [1]. Cachexia has been defined as a multifactorial syndrome characterized by an ongoing loss of skeletal muscle mass (with or without loss of fat mass) that cannot be fully reversed by conventional nutritional support and leads to progressive functional impairment [2]. More recently, cachexia has been conceptualized as a type of disease‐related malnutrition associated with chronic inflammation that should not be perceived as an end stage of malnutrition. There may be multiple pathogenic backgrounds and differences in impact on specific populations (e.g., older patients with cancer) [2]. The conceptual framework for defining cachexia remains limited. A comprehensive definition that incorporates insights from the publications of Evans et al. and Fearon et al. is needed [2, 3]. Arguably, a novel definition of cachexia may also be influenced by successful clinical trials.

Cancer cachexia has been defined as a continuum with three stages of clinical relevance: precachexia, cachexia and refractory cachexia [2]. Not all patients traverse the entire spectrum, and the risk of progression varies based on disease and patient‐based factors as well as on individual treatment response to specific anticancer therapies. In precachexia, early clinical and metabolic signs (e.g., anorexia and impaired glucose tolerance) can precede substantial involuntary weight loss (i.e., loss ≤ 5% of body weight). Cachexia can be diagnosed in patients who have more than 5% loss of stable body weight over the past 6 months, a body mass index (BMI) of less than 20 kg/m^2^ and ongoing weight loss of more than 2% or sarcopenia and ongoing weight loss of more than 2%. The last stage, refractory cachexia, is characterized by a low performance status, together with a very short life expectancy (usually less than 3 months), and is associated with active catabolism or the presence of factors that prevent successful management of weight loss. Cachexia can be clinically refractory as a result of very advanced cancer (preterminal) or the presence of rapidly progressive cancer unresponsive to anticancer therapy [2].

The overall prevalence of cachexia is high and varies according to the underlying condition. Using data from a German cross‐sectional study by Gingrich et al., among 100 older medical inpatients, nearly two‐thirds had at least one of the tissue loss syndromes—sarcopenia, frailty, cachexia or malnutrition—with a significant overlap and interrelationship among them [4]. In a recent scoping review by Ueshima et al. on studies involving Asian adult patients with cachexia due to cancer and chronic diseases other than cancer (e.g., heart failure), the prevalence rates of cachexia were 3.4%–66.2% and 6.2%–93% in noncancer and cancer patients, respectively [5, 6].

During the workshop, experts agreed that for future cachexia trials, body composition should be evaluated, identifying two or three major endpoints, and must involve experienced trialists and biostatisticians. Body weight should be measured twice: at the beginning and end of the trial. Muscle mass (i.e., skeletal muscle mass) should be assessed, but muscle function (e.g., using hand‐grip or stair‐climbing tests) is more relevant and is not necessarily correlated with muscle mass. For instance, decreased gait speed was shown to be a predictor of mortality in several clinical trials; therefore, the quantity, as well as the functional ability of the musculature, is important [7, 8, 9].

## 3 Standard of Care in Cachexia

### 3.1 What Is the Standard of Care in Cachexia?

No consensus exists to date on a single effective or standard treatment for the management of cachexia. Many different disciplines are involved: patient and caregiver education (frequently patients are not aware of their condition), effective antineoplastic therapy (an effective antineoplastic therapy can also improve cachexia outcomes), symptom management (such as pain and palliative care therapies), registered dietitian engagement (e.g., nutritionists), exercise and physiotherapy and investigation of new therapies in clinical trials [10]. Therapeutic interventions that show benefit in cancer cachexia may be effective in other types of cachexia as well. Currently, therapeutic interventions mainly focus on alleviating the consequences and complications of cachexia, e.g., symptom control (appetite stimulation, management of nausea or eating‐related distress of patients and families). Some therapeutic interventions also target muscle mass and/or function. Recently, ponsegromab, a humanized monoclonal antibody inhibiting growth differentiation factor‐15 (GDF‐15), was evaluated in a Phase 2, randomized, double‐blind, 12‐week trial, showing an increase in weight and patients' overall activity level and reducing cachexia symptoms in cancer cachexia [11]. Pfizer, the manufacturer of ponsegromab, is now considering moving to Phase III, and study plans were posted on ClinicalTrials.gov in May 2025, outlining trials that will further assess effects on body weight, physical function and symptom burden (NCT06989437). Still, even though some drugs also reached evaluation in Phase III clinical trials (e.g., the selective androgen receptor modulator enobosarm) [12], none of the drugs currently under evaluation protected adipose tissue [13]. Any cachexia treatment should ideally not result in loss of adipose tissue and may even cause an increase, but clinical trials were rarely adjusted for fat content.

### 3.2 Is It Acceptable to Have No Special Nutritional Support and No Exercise in a Trial Control Group?

The main contributing factors leading to an impairment of nutritional status in cancer cachexia are intestinal malabsorption, reduced bioavailability of nutrients, reduced food intake and increased nutrient loss. These four different features may lead to decreased mobility, increased fatigue and low or poor response to therapy, leading to a further decrease in nutritional status [14]. Prof. Andrew Coats (Sydney Heart Research Institute, Sydney, Australia) addressed the methodological question of whether it is necessary to mandate specific nutritional support and exercise interventions in the control arms of clinical trials for cancer cachexia. He argued that such mandates are neither essential nor practical, primarily because of the current lack of robust evidence defining optimal interventions and the substantial variability in patient needs and applicability and deliverability. Coats emphasized that there is no clear consensus on the precise forms of nutritional support or exercise that would benefit particular patient groups and that setting rigid requirements in trials risks introducing unnecessary complexity without clear therapeutic benefit.

On the other hand, nutritional interventions seek to optimize food intake, preserve or enhance skeletal muscle mass and improve physical performance, thereby mitigating metabolic disturbances and helping patients adhere to their prescribed treatment regimens without dose reductions. Artificial nutrition and supplementation, it was argued, should be implemented when necessary if it is not possible for patients to reach requirements for health and treatment optimization. A recent systematic review of 252 randomized controlled trials (RCTs) involving 31 067 cancer patients, and investigating dietary interventions, showed that most RCTs are small (median sample size of 71; 68% of studies recruited < 100 patients) and measure nonclinical endpoints (only 3% of RCTs evaluated overall survival, 6% of those evaluated quality of life and 8% directly measured cancer) [15]. The few large RCTs conducted to date have not shown an improvement in cancer outcomes. Therefore, there is currently limited evidence to support dietary interventions as a therapeutic tool in cancer care [15]. This lack of strong evidence is reflected in current clinical guidelines. Both the European Society for Medical Oncology (ESMO) and the American Society of Clinical Oncology (ASCO) provide only weak recommendations for dietary counselling, enteral and parenteral nutrition in the context of cancer cachexia management. ESMO classifies its recommendations at Level II, indicative of evidence derived from small randomized trials or trials with methodological limitations [16]. Similarly, ASCO identifies dietary counselling as having moderate benefits with low risk of harm, whereas specialized nutritional support carries low evidence of benefit and a moderate risk profile [17].

### 3.3 What About Exercise Therapy?

Exercise is widely recognized for its general health benefits and shows increasing promise in oncology settings. Still, the current evidence specific to cancer cachexia is inconsistent and incomplete. Prof. Coats noted the absence of clear guidance on optimal exercise modalities, poor patient adherence and the practical challenges of implementing exercise regimens during active cancer treatment. There is a lack of understanding of whether resistance vs. aerobic exercise would be of benefit. It is considered especially difficult to deliver resistance exercise in the elderly (and that will certainly also be the case for patients with cachexia). Existing trials report heterogeneous and inconclusive results in terms of muscle mass, metabolism and physical function. Consequently, there remains an unmet need for further research to establish definitive, mechanistically grounded exercise interventions in this context. Indeed, many pathological changes contribute to muscle atrophy in cancer cachexia: factors related to skeletal and cardiac muscles (myokines and cardiokines); factors secreted by cancer and cancer‐associated immune cells (e.g., transforming growth factor, damage‐associated molecular patterns and leukaemia inhibitory factor), which may trigger a cascade of processes that ultimately result in cachexia; and circulating factors, intracellular signalling pathways and atrophic end effectors, which lead to cardiac and skeletal muscle atrophy [14].

Focusing on skeletal muscle maintenance has the potential to reduce systemic inflammation and improve patients' metabolism and overall physical function. It is clear that physical activity and exercise are beneficial during cancer treatment and survivorship and have clear potential as a nonpharmacological treatment for muscle‐wasting conditions [14]. A recent systematic review of 12 studies (nine randomized and three non‐RCTs), involving a total of 898 patients, identified modest evidence to support the use and continued study of exercise training for the management of cancer cachexia [18]. Exercise interventions appear to be safe in this population, and once enrolled, programs are acceptable to the majority of patients based on adherence and program completion. More consistent positive effects were observed for body composition and muscle strength, as compared with exercise capacity and health‐related quality of life [18]. However, successful RCTs in patients with cancer cachexia and their effects on muscle mass, metabolism and physical function are lacking. Further research is needed to determine the mechanistic basis for these improvements and whether these benefits can be achieved.

Currently, there is a paucity of randomized clinical trials on special nutritional support and exercise therapy. Physical function assessments can be standardized, whereas the voluntary physical activity of patients in trials likely cannot be standardized. It is also difficult to interpret the available randomized clinical trials testing dietary and exercise interventions, mainly because of insufficient blinding and self‐selected enrolment of comparatively more motivated patients. Since dietary and exercise interventions can be very heterogeneous (as can cancer), and results of dietary interventions in one cancer (or one population) may not extrapolate to others, generalizability of the results to a broader population is not always possible. Furthermore, there is a lack of consistency in the guidelines and relatively weak guidelines recommendations. In conclusion, Coats contended that mandating specific nutritional or exercise programs in clinical trials evaluating pharmacological interventions for cancer cachexia is neither justified by the evidence nor operationally feasible. Such mandates could introduce unnecessary variability and noise, potentially confounding trial outcomes. A more pragmatic approach is to conduct trials against the backdrop of standard care appropriate for the patient population, without imposing compulsory interventions that lack robust empirical support.

### 3.4 Nutritional and Symptom Considerations in Patients With Cancer—Anything Special?

Prof. Egidio Del Fabbro (Medical College of Georgia and Study Group of the Multinational Association of Supportive Care in Cancer, MASCC) discussed the evolving understanding of nutritional impact symptoms in cancer cachexia and efforts to systematically address them in clinical guidelines and practice. MASCC, an international organization recognized for its guidelines on mucositis and chemotherapy‐induced nausea and vomiting, is now applying its expertise towards developing recommendations that encompass symptoms affecting nutritional intake. Del Fabbro emphasized that symptoms influencing cachexia extend beyond appetite alone, encompassing a broad array of factors contributing to tissue wasting, including both muscle and fat loss. Accumulating evidence suggests that various symptoms—such as pain, dry mouth, depression and gastrointestinal disturbances—not only impair food intake but are also associated with adverse outcomes like decreased physical activity, malnutrition and reduced survival. Malnutrition with underlying disease develops through two parallel and partly intertwined pathways: In the absence of inflammation, symptoms affecting nutrition (e.g., appetite loss, dysphagia and malabsorption) result in low food intake and reduced assimilation, and with inflammation, processes that lead to muscle and fat breakdown increase in activity and result in tissue loss. Inflammation also often induces loss of appetite that may decrease intake as well [19].

Current recommendations by the European Society for Clinical Nutrition and Metabolism (ESPEN) recommend for oncology patients a target intake of between 25 and 30 kcal/kg/day and 1–1.5 g/kg/day of protein [20]. A retrospective study conducted by Nasrah et al. examined the relationship between weight change and both diet and change in dietary intake in patients with advanced‐stage cancer referred to a multidisciplinary clinic for management of cancer cachexia [21]. It was shown that—at referral—over 25% of patients were consuming a diet that would be inadequate to maintain weight even in healthy adults, and nutritional intake did not reach current recommended levels for energy and protein in over 80% of patients [21]. Furthermore, the change in dietary intake across any visit interval was highly dependent on initial nutritional intake: Thus, those consuming inadequate diets increased their intake significantly more than those already consuming a high‐energy and protein diet. However, even when stratified by initial diet category, there was no consistent correlation between change in diet and change in weight [21]. When patients were categorized by weight change (weight loss > 1 kg, weight stable, weight gain > 1 kg), there was a numerical trend towards a greater increase in energy intake in those who had gained weight, but this did not reach statistical significance [21].

Finally, as mentioned briefly above, symptoms also affect nutrition. As proof of this, a specific term, nutrition impact symptoms (NIS), is frequently used referring to symptoms that compromise food intake and in turn deteriorate nutritional status and drive body weight loss in patients with cancer. As a consequence, it is also clear that although cachexia cannot be corrected with nutritional interventions alone, managing single symptoms (e.g., pain) or aggregate symptom burden can also lead to improved nutritional intake. Studies in cancer patients undergoing surgery with greater NIS were associated with worsened global quality of life, social and physical function at 6 months after surgery for oesophageal cancer, regardless of preoperative BMI or postoperative weight loss [22]. Most of the symptoms can be easily and inexpensively tackled through supportive care [23]. In order to attain nutrition intake goals, an interdisciplinary team should be considered. For instance, psychosocial interventions can mitigate eating‐related distress in patients [24], dietary counselling improves energy intake, body weight and quality of life in radiotherapy [20], whereas nurse‐led interventions mitigate weight and eating‐related distress in patients' caregivers [25]. Another nurse‐led strategy could involve a ‘Walk and Eat’ intervention during chemo‐radiotherapy: A pilot RCT by Xu et al. showed that during chemoradiotherapy, participants who received the walk‐and‐eat intervention had 100‐m less decline than controls in walk distance (adjusted *p* = 0.012), 3‐kg less decrease in hand‐grip strength (adjusted *p* = 0.002) and 2.7‐kg less reduction in body weight (adjusted *p* < 0.001), regardless of age. The intervention group also had significantly lower rates of need for intravenous nutritional support and wheelchair use [26]. Further RCTs are needed to better understand the role of NIS over nutritional intake in patients affected by cancer cachexia.

## 4 Endpoints for Cachexia Trials

### 4.1 Muscle Mass Estimates in Clinical Trials by CT Scan—Is This Precise and Actionable?

Professor Vickie Baracos (University of Alberta, Canada) addressed the precision and clinical actionability of estimating skeletal muscle mass using CT‐derived cross‐sectional imaging, particularly in oncology contexts. Although weight loss is adequate to be included in a cachexia clinical trial, weight loss (not otherwise specified) is insufficient as a criterion of efficacy of a cachexia therapeutic; it should be mandatory to confirm the composition of the weight gain. Building on foundational work by Steven Heymsfield, validating concordance between computed tomography (CT) and magnetic resonance imaging (MRI) in measuring skeletal muscle cross‐sectional area, oncology researchers adopted the approach of analysing the mid‐lumbar (L3) region, which was developed in 2008 and has the strongest correlation with whole‐body muscle volume [27]. This assessment incorporates eight abdominal and paraspinal muscles (rectus abdominis + oblique and transverse abdominis + quadratus lumborum + psoas + paraspinal [multifidus + erector spinae]). It was chosen because cancer imaging is rarely of the whole body, whereas abdomino‐pelvic imaging is relatively common, and the muscle area at L3 has the best correlation with whole‐body muscle mass [28, 29, 30, 31]. Interestingly, this method has gained widespread global use, evidenced by nearly 3000 published studies applying L3 muscle cross‐sectional area analysis across cancer care settings, leveraging routine imaging already performed in clinical practice. Although technically feasible and widely accessible, the application of CT‐based muscle assessment as a clinical trial endpoint remains limited. As of 2024, only eight RCTs involving approximately 800 patients have used lumbar skeletal muscle index (SMI) as a secondary endpoint. For example, the Phase II ponsegromab study reported a 5% increase in lumbar SMI at 12 weeks, with an estimated effect size of 3%–5% across available studies [11]. However, these effect sizes are modest and hover near the threshold of minimum detectable change, thus presenting challenges for robust clinical trial interpretation [11, 32]. Sources of variation in this measurement approach are critical. Instrumental and imaging‐level variability is relatively minor with standard cancer imaging protocols. However, reader precision and biological/clinical variability represent substantial sources of noise. Accurate quantification requires specialized anatomic radiologists, whose intrareader precision (ideally ~1%) determines the least significant change detectable—approximately a 3% difference, if precision is optimal. Decreased reader precision (≥ 1.5%–2%) compromises the ability to reliably detect even modest effect sizes (3%–5%). It is therefore mandatory to address key sources of variability: instrument, imaging level and acquisition parameters, since cancer imaging has to be set to common standards around the world for instruments and protocols; reader precision, involving a specialist in anatomical radiology; and biological/clinical variation, such as individual variability, categorical vs. continuous, effects of cancer therapy and effects of tumour response. Precision reporting is required in muscle cross‐sectional imaging. It is typically determined by reading 30 images × 2 times or 15 images × 3 times, where the least significant change is the smallest difference that can be detected above measurement error [33, 34]. This could also be measured: For instance, to detect changes of the order of ~3%, the reader precision should be within 1.00% [34]. Poorer precision makes it impossible to detect effects of the magnitude that are typically expected with cachexia therapeutics in 12 weeks. On the other hand, biological variation is extensive among patients with identical diagnoses, stage and treatment plan, and should always be considered [35].

### 4.2 A Short History of Trial Endpoints That Could Be Approvable for Cachexia Indications

Prof. Richard Skipworth (Royal Infirmary of Edinburgh/University of Edinburgh, UK) presented a brief history of trial endpoints in cachexia. To obtain regulatory approval for therapeutics targeting cancer‐associated cachexia, he explained, it is generally necessary to demonstrate improvements in at least two of three key domains: form, function and feeling. Historically, many clinical trials have faced difficulties in meeting this threshold, often achieving measurable improvements in form, such as increases in lean body mass, but failing to demonstrate corresponding benefits in either physical function or patient‐reported symptoms. This has led to a pattern of inconclusive results that has hindered drug approvals in this field. A number of trials exemplify these challenges. The POWER trials investigating enobosarm, a selective androgen receptor modulator, demonstrated improvements in lean body mass but failed to show significant changes in stair climb power [12]. Similarly, in the paired Phase III Romana 1 and 2 trials, anamorelin increased lean body mass in patients with cancer‐related cachexia but did not improve hand grip strength [36]. In the subsequent SCALA trials [37], anamorelin led to increased body weight among individuals with advanced non‐small cell lung cancer (NSCLC) and cachexia; however, no significant improvements were observed in the anorexia or cachexia symptom subscores. Notably, despite these limitations, anamorelin was approved in Japan in 2020, based on data from two Phase II studies that demonstrated improvements in lean body mass, body weight and anorexia symptoms, even though no benefit in functional status was observed [38].

To enhance the characterization of cachexia and its heterogeneity, the Cachexia Index (CXI) has been proposed as a novel composite biomarker of the cachectic phenotype. The CXI is calculated as follows:

$$\frac{\left.\text{Skeletal Muscle Index}\;{\left(\mathrm{cm}\right.}^{2}/m^{2}\right)\;x\;\text{Albumin}\;\left(g/\mathrm{dL}\right)}{\text{Neutrophil Lymphocyte Ratio}}$$

This formulation integrates phenotypic wasting, reflected by skeletal muscle depletion, with the underlying etiological driver of systemic inflammation. Such a composite approach is consistent with contemporary definitions of cachexia that emphasize the need to distinguish the syndrome from simple starvation‐type wasting. Cachexia is associated with adverse body composition changes detectable on CT imaging, a reduction in serum albumin and an increase in neutrophil‐to‐lymphocyte ratio; as such, a low CXI is expected in cachectic patients.

A systematic evaluation of endpoints used in cachexia trials has revealed several persistent methodological challenges [39]. Body composition measures, such as body weight and lean body mass assessed by CT or DEXA, are routinely employed and reliably quantified. In contrast, functional endpoints—including hand grip strength, actigraphy‐derived measures and patient‐reported physical function scores (such as those from the EORTC QLQ‐C30)—are underutilized and, when included, have often failed to show statistically significant improvements [40]. Although appetite and dietary intake are central clinical features of cachexia, endpoints measuring these aspects have rarely been used as primary outcomes [41]. Furthermore, quality of life outcomes, when included, have seldom been adjusted for multiple comparisons, posing risks of statistical error that need to be addressed in future research.

An ideal cachexia trial must demonstrate adaptability to various clinical settings and patient populations, while being capable of evaluating both single‐pathway and multi‐pathway therapeutic interventions. It is equally important to define optimal background care strategies that may influence outcomes. Furthermore, the complex interplay between systemic anticancer therapy, tumour progression, cachexia, comorbidities and sociocultural factors must be rigorously considered. Finally, for future success, trials must achieve alignment between a drug's mechanism of action, its therapeutic indication and the selected endpoints to maximize the likelihood of demonstrating meaningful and clinically relevant benefits.

### 4.3 The Voice of the Patient in Assessing Outcomes

Stacie Hudgens (Clinical Outcomes Solutions, University of Illinois, Chicago, IL, USA) provided an overview of patients' needs. She summarized that patient‐reported symptoms associated with anorexia‐cachexia syndrome (ACS) inform a conceptual framework that is critical to consider in clinical development programs. Using a targeted literature review and in‐depth patient interviews conducted between 2017 and 2022, key concepts emerged that are important for assessing the impact of ACS on functional status and health‐related quality of life. Findings from 21 interviews in patients with advanced solid tumours, including those with non‐small cell lung, colorectal and pancreatic cancers, were consistent with existing literature and support the development of a framework aligned with the US FDA Oncology Center of Excellence Patient‐Reported Outcomes Guidance, encompassing disease‐related symptoms, function, tolerability and health‐related quality of life.

Patients consistently reported a loss of appetite, with discussions centring on changes in diet, as well as alterations in taste, smell and texture of food. These changes frequently led to food avoidance and withdrawal from social activities involving meals, such as dining out with family. Patients described how eating became burdensome, with food rendered unappealing or even intolerable. This experience often resulted in significant impacts on emotional and social well‐being, including feelings of isolation and withdrawal from family interactions. Additionally, both sudden and gradual weight loss were commonly reported, with some participants expressing ambivalence; a few indicated that weight loss initially seemed acceptable because they had wanted to lose weight, but they later recognized it as a marker of disease progression.

Physical symptoms described frequently included tiredness, fatigue, loss of energy, nausea, vomiting and generalized weakness. These symptoms were intricately linked to appetite loss and weight changes, compounding patients' functional decline. Importantly, patients articulated that the functional impact of ACS extended beyond what is typically measured in clinical trials. Although measures such as the 6‐min walk test and hand grip strength provide valuable objective data, patients emphasized that their primary concern was maintaining independence in daily tasks. They prioritized activities such as walking from the bedroom to the bathroom, transferring from a bed to a chair or performing basic self‐care tasks like showering and toileting—activities that are fundamental to their sense of autonomy and quality of life.

Functional status, as perceived by patients, is best assessed through direct report or observation of their ability to manage everyday life, including household and work chores, socializing and self‐care. Both patients and caregivers closely monitored food intake, caloric and protein consumption, weight, movement and functional transfers, recognizing that each decrement in physical capacity led to further deterioration in quality of life. Caregivers played an active role in supporting nutritional intake, mobility and functional preservation, underscoring the multidimensional nature of patient‐centred care in ACS.

To be fully patient‐focused, it is imperative that clinical development teams account for how appetite loss and associated weight loss affect patients across all facets of life. Endpoints in clinical trials should move beyond narrow measures of physical function and incorporate broader assessments that capture the ability to maintain daily activities and independence. Ultimately, delaying functional deterioration and supporting everyday capabilities represent outcomes of the greatest relevance to patients and their families living with advanced cancer and cachexia.

## 5 Therapeutic Approaches and Regulatory Issues

### 5.1 MENAC Approach

The Multimodal intervention with Exercise, Nutrition and Anti‐inflammatory Medication (MENAC) has recently been proposed in order to evaluate whether a multimodal intervention targeting key aspects of cachexia will be beneficial. Prof. Barry Laird (University of Edinburgh, UK) explained that this approach includes several different interventions: dietary counselling, in order to increase nutrition; exercise (aerobic and resistance), in order to target muscle anabolism; administration of anti‐inflammatory drugs (such as ibuprofen), in order to downregulate inflammatory response; omega‐3 oral nutritional supplements, in order to both increase nutritional intake and downregulate the inflammatory response; and systemic anticancer therapy, in order to treat the main reason for cachexia (i.e., cancer). A multimodal approach was already proposed by Ken Fearon in 2008, but it was very challenging to pursue [42].

A randomized, open‐label trial of a MENAC approach plus standard care vs. standard care alone to prevent/attenuate cachexia in advanced cancer patients undergoing chemotherapy was recently performed [43]. The primary objective of the MENAC trial was to prevent the development of cachexia and/or to attenuate cachexia progression in high‐risk patients. The primary outcome was the difference in weight change (as it is a key defining factor of cachexia and is meaningful for both patients and clinicians), whereas the secondary outcomes were the difference in muscle mass assessed by CT scans and physical activity assessed with ActivPal (average daily step count). The key eligibility criteria were newly diagnosed Stage 3 or 4 NSCLC or pancreatic ductal adenocarcinoma (PDAC) receiving systemic anticancer therapy. Weight loss was not a prerequisite. Recruitment was from May 2015 to February 2022. In this period, randomized patients were 105 in the intervention arm and 107 in the standard care arm. Lost to follow‐up were 11 for the intervention group and 20 for the standard care group, whereas those analysed for the primary endpoint were 96 for the intervention group and 92 for the standard care group. The MENAC approach was well tolerated. The primary endpoint of the mean weight change [SD] was 0.05 kg [3.8] in the interventional group vs. −0.99 kg [3.2] in the control group; the mean difference in weight change between arms was −1.04 kg (95% confidence interval [CI] −2.02 to −0.06, *p* = 0.04). Regarding the secondary endpoints, muscle mass showed a mean change [SD] of −6.5 cm^2^ [10.1] intervention vs. −6.3 cm^2^ [11.9] control (*p* = 0.93), whereas mean step count showed a mean change [SD] of −378 [2075] for the intervention group vs. −458 [1858] for the control group (*p* = 0.89). There were 28 and 24 reported serious adverse events (SAEs) in the intervention and control arms, respectively, whereas no suspected unexpected serious adverse reactions (SUSARs) were reported [43]. This was the first large trial examining the multimodal hypothesis for cachexia, providing real‐world data in a pragmatic trial on the background of the changing landscape of systemic anticancer therapy, but also providing a background for optimal cachexia care to test new therapies. Conversely, challenges with optimizing compliance and ensuring fidelity still exist.

### 5.2 Endevica Bio Approach

Endevica is engaged in the scientific understanding of the central melanocortin (MC) system. The central MC system plays an essential role in the regulation of appetite, body mass and energy homeostasis and is implicated in several disease areas [44, 45, 46, 47, 48, 49]. Endevica's CMO, Dr. Dan Marks, discovered the link between the MC system and involuntary weight loss [46]. Endevica's peptide engineering safely unlocked the MC as a therapeutic target for multiple indications, including those related to metabolism and weight management. Particularly, it was shown that blockade of the MC‐4 receptor (MC4R) signalling restores normal appetite during sickness [44, 45]. Therefore, Endevica is developing a portfolio of engineered MC agonists and antagonists across a variety of indications, including oncology, diabetes and obesity. In a preclinical study, TCMCB07, a synthetic antagonist of the MC‐4 receptor, was shown to improve appetite, stabilize body weight, preserve fat and heart mass and slightly protect lean mass after multiple cycles of chemotherapy in several rat models, without notable adverse effects or increased chemotherapy‐related toxicities [44]. During Phase 1, B07 induced mass gain with clinically relevant results observed within 1 week: Healthy normal volunteers on B07 gained 1.47 kg more than on placebo and 2.05 kg overall (*p* = 0.0894) over 5 days of dosing 75 mg/day, whereas daily calories went from 2200 on a standard clinical diet to a peak of 3400 for sedentary human healthy volunteers on B07. No clinically significant trends in safety laboratory parameters, vital signs, ECG parameters or histamines were measured. No SAEs related to the administration of B07 were observed, whereas the majority of adverse events were associated with the injection site. In particular, all injection site reactions were Grade 1 (mild) or Grade 2 (moderate) and resolved prior to the end of the study. Furthermore, B07 is a water‐soluble peptide with no CYP induction/inhibition, so minimal drug–drug interaction is expected in patients affected by cancer.

Weight loss both before and during cancer treatment is a prognostic indicator for overall survival, and total mass loss and muscle mass loss are strongly correlated with mortality in cachectic patients [50, 51]. Furthermore, patients with cancer are at risk for malnutrition and involuntary weight loss due to overactivity in the Central MC System [46]. Therefore, early intervention should increase the survival of cancer patients by preventing mass loss. B07 increased appetite and mass gain in preclinical and clinical trials and should improve overall survival. This could lead to a paradigm shift from a ‘traditional approach’, in which cachexia is treated upon diagnosis in a reactive manner, to a ‘prophylactic approach’, in which cachexia is proactively prevented with adjuvant treatment to maintain patients at a healthy body weight, preventing weight loss and muscle wasting and leading to optimal patient survival, quality of life and function.

### 5.3 Artelo Approach

Prof. Barry Laird continued to present data on ART27.13, a fully synthetic potent cannabinoid (CB) dual CB1/CB2 receptor agonist, originally developed by AstraZeneca and designed to mimic the effects of tetrahydrocannabinol (THC) but not to cross into the brain, thus reducing side effects. The mechanism by which ART27.13 has its effects on appetite and metabolism is driven through the peripheral CB1 receptor [52]. This can be direct stimulation of the tissue, the release of endocrines and other signalling molecules and by direct stimulation of the vagus nerve back to the brain. An experiment conducted by Artelo showed that ART27.13 may also have some direct effects on protecting human muscle tissue from the toxic effects of cytokines and inflammatory molecules produced by tumour cells by acting on the CB2 receptors, which are largely implicated in immune and inflammatory responses [53]. Evidence from the multiple ascending dose study suggests a dose‐dependent increase in body weight [52].

These effects are being evaluated in the Phase 1 and 2 Cancer Appetite Recovery Studies (CAReS) by evaluating weight gain, appetite, inflammatory markers and activity, which we hope will translate into an improved quality of life for cancer patients. In the CAReS Phase 1b Study [54], 27 patients with incurable cancer, weight loss > 5% in the previous 6 months and reduced appetite were enrolled to determine the safety profile of ART27.13 at different doses, dose‐limiting toxicity, if any, within the range of doses used during the first cycle after the first dose of ART27.13, and the most effective, safe dose (recommended Phase 2 dose, or RP2D) to be used as a starting dose in Stage 2. Secondary objectives were body weight, appetite, quality of life and body composition. Doses explored ranged from 150 to 650 μg. There were no events considered as dose‐limiting toxicities and no fatal adverse events related to trial treatment. The most common adverse events (> 1 patient) related to trial drug were somnolence (11%; 3 of 27 patients) and dry mouth (11%). No dose response for adverse events was noted. Furthermore, ART27.13 was well‐tolerated up to 650 μg per day for 12 weeks, with no SAEs attributable to the investigational drug in patients suffering from anorexia associated with cancer. Impact on weight gain or stabilization was observed in two‐thirds of patients [54]. The CAReS Phase 2a study is ongoing and currently open in the United Kingdom, Ireland and Norway at 14 sites (multiple sites in each country). It is exploring doses of up to twice that evaluated in the Phase 1b—max of 1300 μg—in patients with all cancer types (including haematological), not on systemic anticancer therapy, and with Karnofsky Performance Status > 50. The primary outcome measures are change in lean body mass determined by weight and dual‐energy X‐ray absorptiometry (DEXA) body scans at Week 12 and the change in anorexia determined by visual analogue scale at baseline, Week 4, Week 8, Week 12 and at the 30‐day follow‐up visit. Secondary outcome measures are weight, quality of life and activity. Further evidence from the CAReS Phase 2a will help to clarify the efficacy and safety of ART27.13 in a cohort of all types of cancer patients [54].

### 5.4 Actimed Approach

The primary driver of cachexia is an imbalance between catabolism (the breakdown of tissues such as muscle or fat) and anabolism (the build‐up of tissue). This imbalance occurs against a backdrop of poor appetite and reduced food intake in many patients, as well as a general state of inflammation [55, 56, 57]. S‐pindolol represents the first oral anabolic‐catabolic transforming agent (ACTA), which targets the imbalance in catabolism and anabolism by increasing appetite and reducing fatigue via central serotonin 1A (5‐HT_1A_) activity, correcting disease‐driving metabolic imbalance and thereby saving muscle and fat tissue, but also reducing catabolism via β_1_‐blockade and increasing anabolism via partial β_2_‐agonism [55, 56]. The S‐pindolol for the treatment and prevention of cachexia in patients with Stage III/IV NSCLC or colorectal cancer: A randomized, double‐blind, placebo‐controlled, international multicentre Phase II study (ACT‐ONE) trial evaluated two different doses of S‐pindolol (high‐dose S‐pindolol 10 mg bd and low‐dose S‐pindolol 2.5 mg bd) vs. placebo in 87 patients affected by colorectal cancer or NSCLC and cancer cachexia [56]. The primary outcome measure was the difference in the rate (slope) of weight change between high‐dose S‐pindolol and placebo, whereas the main secondary outcomes were body composition, functional parameters (e.g., hand grip strength), the comparison between high vs. low dose and safety. High‐dose S‐pindolol produced a statistically and clinically significant weight gain (+0.54 kg/4 weeks, 95% CI 0.38–0.70) compared with a weight loss on placebo (−0.21 kg/4 weeks, 95% CI −0.37–0.05; *p* < 0.0001). High‐dose S‐pindolol produced a statistically significant increase in lean body mass (*p* = 0.012), whereas changes in fat mass were neutral. Hand‐grip strength significantly (high dose −1.15 ± 0.7 kg, placebo −3.51 ± 0.8 kg change per 4 weeks; *p* = 0.0134), stair climbing power and 6‐min walk test non‐significantly were all directionally in favour of high‐dose S‐pindolol. There were no clinically significant differences in safety signals or survival between treatment groups, although a numerical excess of dyspnoea was seen with high‐dose S‐pindolol (19.1%) compared with placebo (3.2%) [56]. Large treatment effect and favourable safety profile encouraged further clinical investigations with S‐pindolol for the treatment of cancer cachexia. A recent comparative Phase I bioavailability study of S‐pindolol benzoate vs. racemic pindolol showed no statistical differences in blood pressure (either systolic or diastolic blood pressure), with significant increase of heart rate in racemic pindolol group at Day 4 vs. baseline (*p* = 0.0028) and significant increase in heart rate in racemic pindolol group vs. S‐pindolol at Day 4: racemic pindolol vs. S‐pindolol 5 mg (*p* = 0.044); racemic pindolol vs. S‐pindolol 10 mg (*p* = 0.014); and racemic pindolol vs. S‐pindolol 15 mg (*p* = 0.046) [58]. There is also no stereo conversion of R‐pindolol to S‐pindolol in vivo. The IMProving cancer cachexia with ACTAs (IMPACT) program refers to two planned Phase IIb/III studies, aiming to randomize about 300–350 patients in each study to show efficacy in two cancer cachexia indications (as already studied in ACT‐ONE). Primary outcome measures will be body weight, functional assessment and patient‐reported outcomes. The main secondary outcomes will be functional parameters, quality of life and safety [59]. In addition, there will be a pooled analysis of all recruited patients (to study all‐cause mortality of patients together with the PRO outcomes as well as by itself).

## 6 Conclusions

Cachexia, a syndrome marked by weight loss, muscle wasting and functional decline, affects 50%–80% of cancer patients and worsens quality of life, treatment tolerance and survival. Despite its impact, there are no widely approved therapies outside Japan (where anamorelin is approved), with past trials often hampered by complex disease mechanisms and limited efficacy of available interventions. Therapies such as nutritional supplements, progestins, anti‐inflammatories, anabolic agents and newer candidates like anamorelin have shown mixed results, often improving appetite or lean mass without consistent functional benefit. Measuring muscle mass by CT scans at the L3 vertebra is widely used and correlates with whole‐body muscle mass, but small effect sizes and biological variability limit its value as a standalone endpoint. Trials have struggled to meet regulatory standards requiring benefits in both body composition and function or symptoms, with functional measures like grip strength and actigraphy underused and appetite or quality‐of‐life metrics rarely prioritized. The summary of important regulatory discussion points is reported in Table 1. Patients emphasize maintaining independence and daily activity over isolated physical measures, underscoring the need for endpoints that capture real‐life functioning and social participation, with caregivers also playing a key role in monitoring nutrition and mobility [60, 61].

**TABLE 1:** Summary of important regulatory discussion points.

Important regulatory discussion points
1. Clinically meaningful changes in PROs, physical function and/or morbidity/mortality need to be shown to document clinical efficacy for regulatory approval.
2. Reasonable amount of safety needs to be documented: Typically, 300–500 patients with at least 6 months and 100 patients with at least 12 months of follow‐up in cancer cachexia.
3. PROs need to be validated instruments in the underlying (chronic) illness.
4. Avoid asking too many questions in patients with very advanced disease.
5. Hand grip strength and weight changes alone are not sufficient for regulatory approval.
6. In cancer cachexia, to get a ‘pan‐cancer’ indication, one needs to show benefits in 3 distinct cancers (or maybe in 2 of them plus in one ‘basket study’).
7. General physical activity data may be of interest, if the tool is providing valid data.
8. Mode of action data (for instance, by showing body composition changes) would be good to have, but appears not mandatory: The key is whether treatments are beneficial for patients.

Abbreviation: PROs = patient‐reported outcomes.

The MENAC trial tested a multimodal intervention combining nutrition, exercise, anti‐inflammatory drugs, omega‐3 supplements and cancer therapy in patients with advanced lung or pancreatic cancer. It showed a modest benefit in weight stabilization but no significant improvements in muscle mass or activity, demonstrating the feasibility of complex interventions but highlighting challenges in adherence and trial execution. Endevica Bio is advancing TCMCB07, an MC‐4 receptor antagonist that restored appetite, stabilized weight and preserved fat and heart mass in chemotherapy‐exposed animal models without notable side effects. In a Phase 1 trial, B07 led to clinically relevant weight gain and increased caloric intake with good tolerability, minimal drug interactions and no SAEs, supporting its potential as a prophylactic strategy to prevent weight and muscle loss early in cancer treatment.

Artelo's ART27.13, a peripherally acting dual CB1/CB2 receptor agonist, showed appetite stimulation, weight stabilization and potential muscle protection in preclinical and early‐phase studies without central nervous system side effects. In the Phase 1b CAReS study of 27 cancer patients with cachexia, ART27.13 was well tolerated up to 650 μg daily, with weight gain or stabilization in two‐thirds of patients. An ongoing Phase 2a trial is evaluating higher doses in a broader cancer population to assess efficacy in lean body mass, appetite and quality of life. Actimed's S‐pindolol, the first ACTA, modulates appetite and metabolism through serotonin receptor activity and β‐receptor modulation to preserve muscle and fat tissue. A large Phase IIb/III IMPACT program is planned to confirm benefits on weight, function and patient‐reported outcomes in approximately 700 patients.

Future cachexia trials must align therapeutic mechanisms, supportive care strategies and patient‐centred endpoints that reflect meaningful daily functioning to demonstrate clear and meaningful clinical benefit.

## Conflicts of Interest

F.F. has nothing to declare. S.v.H. reported receiving grants from CTC North, Pharmacosmos, the Innovative Medicines Initiative, AstraZeneca, Amgen, IMI, and the German Center for Cardiovascular Research and receiving personal fees from Vifor, Pharmacosmos, AstraZeneca, Bayer, Boehringer Ingelheim, Pfizer, Edwards Lifesciences, Thermo Scientific BRAHMS Biomarkers, LumiraDx, Novartis, Novo Nordisk, and Merck Sharp and Dohme. A.J.S.C. reported receiving personal fees from Actimed Therapeutics, AstraZeneca, Bayer, Boehringer Ingelheim, Edwards Lifesciences, Eli Lilly, GSK, Menarini, Novartis, Novo Nordisk, Servier, CSL Vifor, Abbott, Actimed Therapeutics, Cardiac Dimensions, Corvia, CVRx, Enopace Biomedical Ltd, ESN Cleer, Faraday Pharmaceuticals, Impulse Dynamics, Respicardia, and Viatris. J.B. is a consultant for Abbott, Adaptyx, American Regent, Amgen, AskBio, AstraZeneca, Bayer, Boehringer Ingelheim, Boston Scientific, Bristol Myers Squibb, Cardiac Dimensions, Cardior, CSL Vifor, CVRx, Cytokinetics, Daxor, Diastol, Edwards, Element Sciences, Faraday, Idorsia, Impulse Dynamics, Imbria, Innolife, Intellia, Inventiva, Levator, Lexicon, Eli Lilly, Mankind, Medtronic, Merck, New Amsterdam, Novartis, Novo Nordisk, Pfizer, Pharmacosmos, Pharmain, Prolaio, Pulnovo, Regeneron, Renibus, Reprieve, Roche, Rycarma, Saillent, Salamandra, Salubris, SC Pharma, SQ Innovation, Secretome, Sequanna, Transmural, TekkunLev, Tenex, Tricog, Ultromic, Vera, Zoll. E.D.F. has nothing to declare. R.J.E.S. has received personal fees for consultancy from Artelo, Actimed, Faraday, and Helsinn. B.J.A.L. has received personal fees for consultancy from Artelo, Actimed, Faraday, Kyona Kirin, Helsinn, and Toray. S.D.A. reported receiving grants from Abbott Laboratories; receiving personal fees from Actimed Therapeutics, Alleviant, AstraZeneca, Bayer, Berlin Heals, BioVentrix, Boehringer Ingelheim, Brahms, Cardiac Dimensions, Cardior Pharmaceuticals GmbH, Cordio, CSL Vifor, CVRx, Cytokinetics, Edwards Lifesciences, Faraday Pharmaceuticals, GSK, HeartKinetics, Impulse Dynamics, Lilly, Mankind Pharma, Medtronic, Novartis, Novo Nordisk, Occlutech, Pfizer, Regeneron, Relaxera, Repairon GmbH, SCIRENT Clinical Research and Science, Sensible Medical, Servier, Vectorious Medical Technologies, Vivus, and V‐Wave; and being a named co‐inventor of 2 patent applications regarding midregional proatrial natriuretic peptide, but does not benefit personally from the related issued patents.
